# Effects of SCCO_2_, Gamma Irradiation, and Sodium Dodecyl Sulfate Treatments on the Initial Properties of Tendon Allografts

**DOI:** 10.3390/ijms21051565

**Published:** 2020-02-25

**Authors:** Yikan Sun, Vedran Lovric, Tian Wang, Rema A. Oliver, William R. Walsh

**Affiliations:** Surgical and Orthopedic Research Laboratories, Prince of Wales Clinical School, Prince of Wales Hospital, University of New South Wales, Randwick, New South Wales 2031, Australia; yikan.sun@student.unsw.edu.au (Y.S.); vedran.lovric@unsw.edu.au (V.L.); t.wang@unsw.edu.au (T.W.); rema.oliver@unsw.edu.au (R.A.O.)

**Keywords:** Allograft, decellularization, sterilization, supercritical carbon dioxide, supercritical fluid, tendon

## Abstract

Sterile and decellularized allograft tendons are viable biomaterials used in reconstructive surgeries for dense connective tissue injuries. Established allograft processing techniques including gamma irradiation and sodium dodecyl sulfate (SDS) can affect tissue integrity. Supercritical carbon dioxide (SCCO_2_) represents a novel alternative that has the potential to decellularize and sterilize tendons with minimized exposure to denaturants, shortened treatment time, lack of toxic residues, and superior tissue penetration, and thus efficacy. This study attempted to develop a single-step hybrid decellularization and sterilization protocol for tendons that involved SCCO_2_ treatment with various chemical additives. The processed tendons were evaluated with mechanical testing, histology, scanning electron microscopy (SEM), and Fourier-transform infrared (FTIR) spectroscopy. Uniaxial mechanical testing showed that tendons treated with SCCO_2_ and additive NovaKill^TM^ Gen2 and 0.1% SDS had significantly higher (*p* < 0.05) ultimate tensile stress (UTS) and Young’s modulus compared to gamma-irradiated and standard-SDS-treated tendons. This was corroborated by the ultrastructural intactness of SCCO_2_-treated tendons as examined by SEM and FTIR spectroscopy, which was not preserved in gamma-irradiated and standard SDS-treated tendons. However, complete decellularization was not achieved by the experimented SCCO_2_-SDS protocols used in this study. The present study therefore serves as a concrete starting point for development of an SCCO_2_-based combined sterilization and decellularization protocol for allograft tendons, where additive choice is to be optimized.

## 1. Introduction

Tendinous and ligamentous injuries are extremely common, especially among at-risk population subgroups such as military recruits, recreational and elite runners, and professional athletes, and contribute to much morbidity and functional loss [1,2,3,4]. The reported incidence rates of anterior cruciate ligament (ACL) injuries that require surgical reconstruction, for instance, are up to 0.52 cases per 1000 person-years in Australia and climb to as high as 1.52–37.62 cases per 1000 person-years for professional athletes [5]. Autogenous tendons are currently considered the gold standard for grafting of large defects [6]. However, they are associated with limited availability, prolonged surgical and rehabilitation time, donor site morbidity, and potential functional deficits [7,8,9,10,11,12]. Use of allografts, on the other hand, circumvents the above-mentioned drawbacks associated with autograft harvesting and implantation, but is discouraged by higher costs, the immunogenicity of the donor cellular materials [13], and most notably, the much-dreaded risk of disease transmission [14,15,16,17,18]. Consequently, in addition to routine donor screening and adherence to aseptic harvesting and handling techniques [19], allograft tendons are recommended to be terminally sterilized to achieve a sterility assurance level (SAL) of 10^−6^ [20], which denotes a 1 in 100,000 probability of any micro-organisms surviving the treatment [21,22,23].

Currently, gamma irradiation stands as the mainstay for musculoskeletal allograft sterilization [24,25,26], which inactivates pathogens by damaging the nucleic acids through direct energy deposition and generation of free radicals [27,28]. Nevertheless, it undermines the biomechanical and biological properties of allograft tendons in a dose-dependent manner [29,30,31,32], which is thought to be mediated by the irradiation-induced free radicals cross-linking collagen [33]. Consequently, controversy persists regarding whether the routinely used moderate-dose 25 kGy gamma irradiation to achieve acceptably low risk of transmission markedly compromises the initial mechanical properties of allograft tendons [31,34,35,36,37,38,39,40,41,42,43,44,45,46,47].

**Figure 1:**
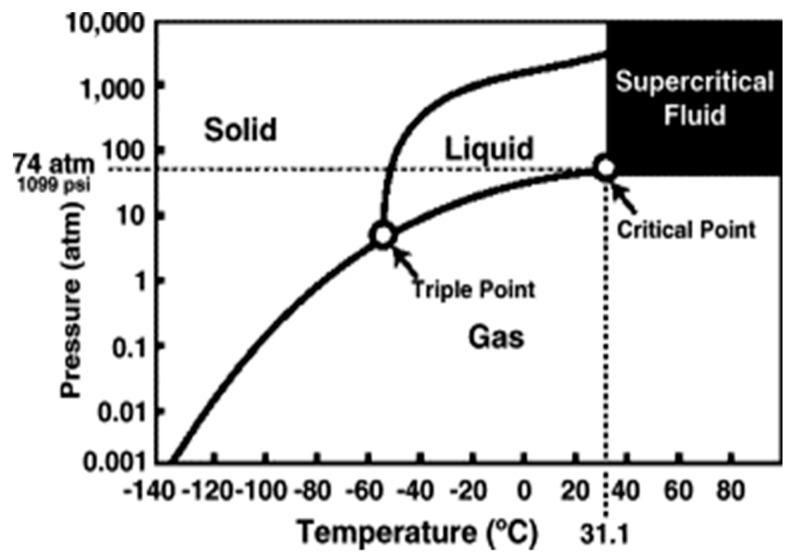
Phase diagram of CO_2_ showing the critical point at 31.1 °C and 1099 psi [54].

Contrarily, supercritical carbon dioxide (SCCO_2_), which has many applications in the food and pharmaceutical industries [48,49,50], emerges as a promising alternative that possesses many properties of an ideal sterilant for biomaterials. As CO_2_ enters its supercritical state at 31.1 °C and 1099 psi (Figure 1), its gas-like viscosity and diffusability allow it to readily permeate the tissue matrices, while its liquid-like density and solvation power [51,52,53] enable synergy with modifier solutions to improve sterilization efficacy [54,55,56,57]. The low critical temperature of CO_2_ makes it suitable for treating heat-sensitive biomaterials by not denaturing the protein content of the tissue [57,58,59,60]. SCCO_2_ can be easily withdrawn without toxic residues from treated biomaterials simply through depressurization and out-gassing [55,61]. Besides, CO_2_ is non-toxic, non-inflammable, and chemically inert, and thus does not constitute an environmental or occupational hazard [62,63]. Meanwhile, its natural abundance and recyclability guarantee the economic feasibility of industrial-scale CO_2_-based sterilization [49,64].

SCCO_2_ has been demonstrated to inactivate viruses [65,66,67], vegetative bacteria [57,62,68], and, working synergistically with additives such as peracetic acid (PAA) and hydrogen peroxide (H_2_O_2_), bacterial spores [69,70] to achieve SAL 10^−6^ [54,61], though the exact mechanism remains obscure [71]. Pioneering studies have found it to be a gentle agent that appears to preserve the mechanical properties of a variety of tissues such as heart valves [59], lungs matrices [72], amniotic membrane [73], bones [66,74,75,76,77], acellular dermal matrices [69], and menisci [78], while there is still a paucity of literature on tendons [40,46,79].

Donor cells and residual DNA have been shown to induce prolonged adverse host inflammatory reactions against allograft tendons in vivo that can lead to clinical inferiority to autografts [13]. Therefore, decellularization, the process of removing resident cells and nuclear material, has been increasingly recognized as a crucial step in the tissue engineering process to mitigate their antigenicity. Decellularization usually entails agitated wash in protein-denaturing detergents followed by prolonged washing with phosphate-buffered saline (PBS) or deionized water to unload toxic residues introduced [80]. It is proposed that SCCO_2_ may be equally, if not more, effective at removing chemical residues while drastically expediating the washing process owing to its solvent properties, diffusibility, and ease of withdrawal from tissue, as described above. Indeed, Casali et al. [81] have recently successfully purged cytotoxic detergent from porcine aortas using SCCO_2_ following solution-based decellularization treatment, shortening the final cleansing step to merely one hour, which typically lasts more than 24 h with saline wash. Additionally, similar to how it drastically improves the sterilization efficacy of PAA while minimizing the chemical concentration required [54], SCCO_2_ has the potential to directly synergize with additive decellularizing detergents such as sodium dodecyl sulfate (SDS) to remove the immunogenic cellular materials, but to our knowledge, such a possibility has not yet been extensively explored by published studies.

SCCO_2_ can potentially engender a single-step protocol with shortened treatment time that produces sterile and decellularized tendon allografts without compromising their biomechanical properties. The aims of the present study was therefore to (a) test if the established SCCO_2_ sterilization protocol preserves the initial biomechanical properties of tendons; (b) histologically assess if SCCO_2_ is able to preserve the morphology of, and decellularize tendons with appropriate additive chemicals; and, in addition, (c) microscopically and molecularly characterize tendons undergoing different treatments through field-emission scanning electron microscopy (FE-SEM) and Fourier-transform infrared (FTIR) spectroscopy, respectively. It was hypothesized that SCCO_2_ treatment would not affect the initial biomechanical properties, ultrastructure, and chemical signatures of tendon grafts and would be able to decellularize tendons with appropriate additives.

A total of 6 groups were proposed for the study: (I) Untreated control; (II) Moderate-dose gamma irradiation (~25 kGy); (III) Standard SDS [82]; (IV) SCCO_2_ treatment with the sterilising NovaKill^TM^ Gen2 additive (SCCO_2_-NK); (V) SCCO_2_ with NovaKill^TM^ Gen2 and 0.1% SDS (SCCO_2_-0.1%-SDS); and (VI) SCCO_2_ with NovaKill^TM^ Gen2 and 1% SDS (SCCO_2_-1%-SDS).

## 2. Results

### 2.1. Gross Appearance and Dimensions

Compared to native tendons, gamma-irradiated tendons became shriveled and their surface granular; the SCCO_2_-NK-treated tendons became noticeably cleaner with effacement of the surface marks made on them prior to treatment; and SCCO_2_-SDS-treated tendons, especially the SCCO_2_-0.1%-SDS treated ones, turned out to be visibly dehydrated, to the extent that they were appreciably transparent, and only regained the typical white and glossy texture of tendons after immersing in PBS for at least 15 min. No perceptible change in the outward appearance was observed for the standard-SDS-treated tendons.

The mean pre- and post-treatment cross-sectional area (CSA) and thickness of the standard SDS group were significantly greater than those of the control group (*p* < 0.05), while the mean post-treatment CSA and thickness of SCCO_2_-0.1%-SDS group were significantly smaller than those of the Standard SDS group (*p* < 0.05). No significant differences were found between the pre- and post-treatment dimensions of tendons in all groups except the SCCO_2_-0.1%-SDS group, where there was a statistically significant decrease in CSA (*p* = 0.020) and thickness (*p* = 0.008), which was in concordance with their dehydrated appearance post-treatment (Table 1).

### 2.2. Mechanical Testing

In general, the mechanical properties of all treatment groups apart from SCCO_2_-1%-SDS were greater than the control group. This may reflect a potential drying artefact that may have accounted for this increase. All tendons failed at the mid-substance and were included in the statistical analysis. SCCO_2_-0.1%-SDS-treated tendons were found to be significantly stiffer than the native tendons (*p* = 0.014) and the standard-SDS-treated tendons (*p* = 0.003) (Table 2a). The mean ultimate tensile stress (UTS) of the standard SDS group was significantly lower than that of the gamma group (*p* = 0.006), and reduced compared to that of the control, although statistical significance was not reached (*p* = 0.062); whereas the mean UTS of the SCCO_2_-0.1%-SDS group was significantly greater than those of the control (*p* = 0.011), gamma (*p* = 0.011), and standard SDS (*p* < 0.001) groups. The mean failure strain of the SCCO_2_-0.1%-SDS was significantly lower than that of the standard SDS group (*p* = 0.035). The mean Young’s modulus of the SCCO_2_-0.1%-SDS group was significantly higher than those of the control (*p* < 0.001), gamma (*p* < 0.001), standard SDS (*p* < 0.001), and SCCO_2_-NK (*p* = 0.019) groups, while the mean Young’s modulus of the SCCO_2_-NK group was also significantly greater than that of the standard SDS group (*p* = 0.003).

However, with a 10-fold increase in concentration of additive SDS from 0.1% to 1%, the SCCO_2_-1%-SDS-treated group had significantly decreased stiffness than the SCCO_2_-0.1%-SDS group (*p* = 0.004), significantly decreased UTS than the control (*p* < 0.001), SCCO_2_-NK (*p* = 0.001), gamma (*p* < 0.001), and SCCO_2_-0.1%-SDS (*p* < 0.001) groups, and significantly reduced Young’s modulus than the SCCO_2_-NK (*p* = 0.033) and SCCO_2_-0.1%-SDS (*p* < 0.001) groups (Table 2b).

Notably, there were no significant differences detected between the control and gamma irradiation groups in regards to the mechanical endpoints analyzed.

### 2.3. Histomorphology

**Figure 2:**
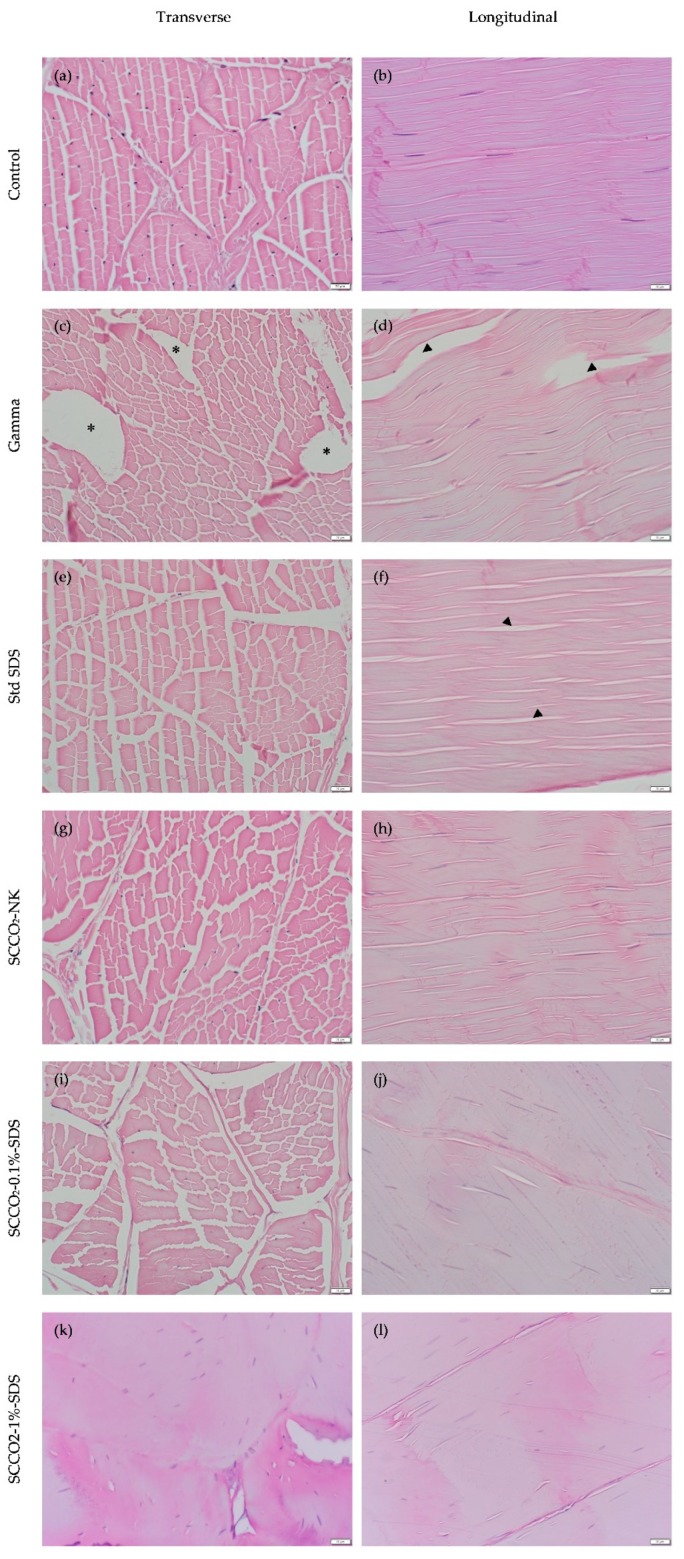
Representative high-resolution histological images of transverse and longitudinal sections. In haematoxylin and eosin (H&E) sections, collagen was stained pale pink with interspersed dark blue nuclei conspicuous in all groups except the standard SDS group. The histological dead space in the transverse sections are indicated with *. Separation of collagen bundles are indicated with black arrowheads. Scale bar at the bottom right represents 20 μm.

The morphology of the extracellular matrix (ECM) was examined and cellularity assessed (Figure 2).

Gamma irradiation and standard SDS treatment both inflicted substantial damage to the ECM of tendons (images c, d, e, and f, Figure 2), as evidenced by perceptible differences in histomorphological appearances in comparison to control (images a and b, Figure 2). Specifically, irregularly-shaped dead space devoid of pink-staining ECM and separation of the collagen fibers were notable in the transverse and longitudinal sections of the gamma-irradiated tendons (images c and d, Figure 2). The increased gaps between collagen fibers in the longitudinal sections were also observed in the standard SDS group (image f, Figure 2), albeit to a lesser extent. These findings on microscopic observation were in keeping with the quantitative results, where there was a significantly increased percentage of void (area not occupied by ECM) in gamma-irradiated (41.81 ± 4.50%) and standard-SDS-treated (35.21 ± 4.48%) tendons than in native tendons (29.08 ± 4.96%) (*p* < 0.001 and *p* = 0.015, respectively) (Table 3).

Conversely, no remarkable morphological changes of collagen fibers were noted for the SCCO_2_-NK or SCCO_2_-0.1%-SDS-treated specimens (images g, h, I, and j, Figure 2), as evidenced by the quantitative results that SCCO_2_-NK-treated tendons have significantly less percentage of void (27.61 ± 6.49%) compared to gamma-irradiated (41.81 ± 4.50%) and standard-SDS-treated (35.21 ± 4.48%) ones (*p* < 0.001 and *p* = 0.001, respectively), and so do SCCO_2_-0.1%-SDS-treated tendons (27.35 ± 7.09%) (*p* < 0.001 and *p* = 0.001 when comparisons made to gamma-irradiated and standard-SDS-treated tendons, respectively). Interestingly however, the spaces between the collagen fibers as observed in the transverse and longitudinal sections of the native tendons appeared to be nearly obliterated by the SCCO_2_-1%-SDS treatment (images k and l, Figure 2), which was corroborated by drastically decreased empty space on quantitative analysis (7.48 ± 5.18%) (Table 3).

Only the standard SDS treatment achieved thorough decellularization with near absence of distinguishable cell nuclei histologically (3.17 cells/mm^2^). The cellularity of SCCO_2_-0.1%-SDS-treated tendons (682.32 ± 126.42 cells/mm^2^) was significantly lower than that of the control tendons (818.91 ± 116.62 cells/mm^2^) (*p* = 0.021), but this reduction is trivial when compared to the absolute number of cells left within the tendons (Table 3).

### 2.4. Scanning Electron Microscopy

**Figure 3:**
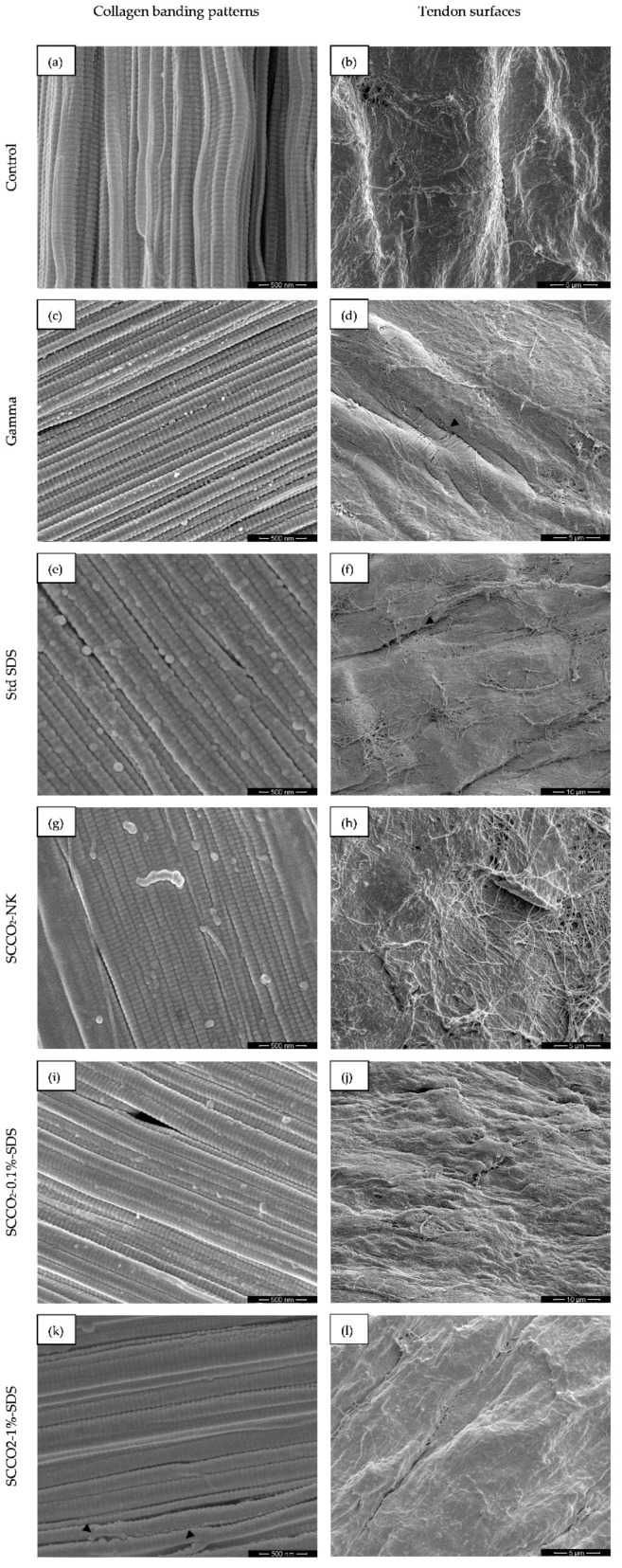
The typical appearances of the collagen banding patterns as observed under ~100,000× magnification (left column, **a**,**c**,**e**,**g**,**i**,**k**) and the tendon surfaces as observed under ~10,000× magnification (right column, **b**,**d**,**f**,**h**,**j**,**l**). Black arrowheads in the left column indicate breaks in collagen fibers and those in the right column indicate sites of surface breakage and exposure of underlying collagen fibrils.

Tendon surfaces and the collagen triple helices running in the longitudinal direction were observed under high magnification (Figure 3).

Under approximately 10,000× magnification, the surfaces of SCCO_2_-NK-, SCCO_2_-0.1%-SDS-, and SCCO_2_-1%-SDS-treated tendons (images h, j and l, respectively, Figure 3) resembled that of the native tendons (image b, Figure 3), whereas gamma-irradiated and standard-SDS-treated tendons (images d and f, respectively, Figure 3) exhibited numerous fissures on their surface, exposing the collagen fibers underneath.

Under ultrahigh (~100,000×) magnification, disruption to the collagen banding pattern was appreciable in all treated tendons, especially the standard-SDS- and SCCO_2_-1%-SDS-treated ones (images c and k, respectively, Figure 3). Specifically, for SCCO_2_-1%-SDS treated tendons, the surface of the collagen fibers appeared granular and occasional breaks in fibers were readily identifiable. Meanwhile, standard SDS treatment caused destructive changes to the characteristic collagen triple helices, where innumerable bulges annihilated the smooth cylindrical contour seen in the pristine fibers of control tendons (image a, Figure 3).

### 2.5. Fourier-Transform Infra-Red Spectroscopy

**Figure 4:**
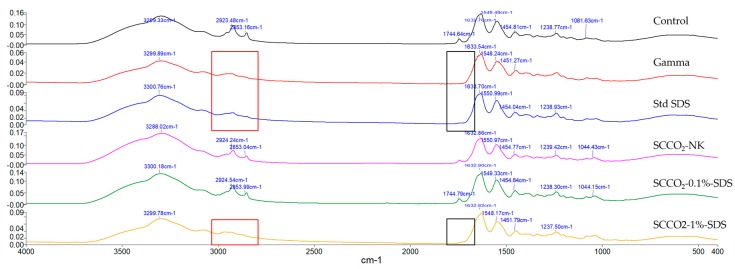
Representative FTIR spectra of the control, gamma-irradiated, standard-SDS-treated, SCCO_2_-NK-treated, SCCO_2_-0.1%-SDS-treated, and SCCO_2_-1%-SDS-treated tendons. The red and black boxes highlight the reductions of absorption peaks associated with CH_2_ and carbonyl groups, respectively.

The FTIR spectra of short tendon segments yielded valuable insights into their spatial molecular composition (Figure 4). No significant shifts or absolute reductions in absorption peaks, including primarily the amide I (1634 cm^−1^), II (1548 cm^−1^), and III (1250 cm^−1^) peaks associated with collagen [83,84], were detected in the SCCO_2_-NK- and SCCO_2_-0.1%-SDS-treated samples, indicating no or minimal damage to the collagen contents. Also, the shapes of the spectra of native, SCCO_2_-NK- and SCCO_2_-0.1%-SDS-treated tendons between 1125 and 970 cm^−1^ are strikingly similar, implying preservation of various other ECM components such as proteoglycans [85].

Conversely, standard SDS and gamma irradiation appeared to have obliterated the peaks at around (a) 2920 cm^−1^ and 2850 cm^−1^, which correspond to, respectively, the asymmetric and symmetric stretching of the collagen-associated CH_2_ group; and (b) 1742 cm^−1^, which is associated with the carbonyl group [86]. SCCO_2_-1%-SDS treatment provoked a similar pattern of absorption peak changes.

## 3. Discussion

Decellularization and terminal sterilization are crucial steps in soft tissue allograft manufacturing. In principle, any viable constituents should be removed, or at least deactivated, in the end-product, and the ECM ideally conserved in its intact three-dimensional microarchitecture, which contains naturally occurring collagen, and is responsible for most of its mechanical robustness [87,88]. Tissues are processed in this manner so that the risk of disease transmission is minimized with the removal of pathogens, adverse host response avoided in the absence of cellular materials, and graft incorporation promoted owing to the capability of intact ECM to induce healing and remodeling [80]. However, concerns have been raised regarding the harmful effect of the currently used physical and chemical processing techniques on the biomechanical properties on allograft tendons, which might have been implicated in their suboptimal clinical performance and propensity to fail.

Our results suggest that the SCCO_2_-based NovaKill sterilization protocol, even with the addition of 0.1% SDS to the antimicrobial cocktail, was able to preserve the mechanical integrity of the tendons, as they showed similar if not greater level of UTS and Young’s modulus compared to the untreated tendons. This finding largely coincides with the results of the few previously published studies on SCCO_2_-treated tendons and collagenous soft tissues, where they were consistently found to have comparable or increased UTS to the untreated tendons [40,46,59,79]. It should also be noted that SCCO_2_-0.1%-SDS-treated tendons were biomechanically superior to the standard-SDS-treated tendons with respect to UTS and Young’s modulus, even though the standard SDS protocol re-performed in the present study also employed the same concentration of SDS (0.1%) and was the least destructive one to accomplish decellularization among the trialed protocols in the original study conducted by Pridgen et al. [82]. Admittedly, although it is capable of lysing cell and nuclear membranes, SDS along with some other commonly used decellularizing chemicals, such as tri(n-butyl)phosphate (TnBP) and Triton X-100, is notoriously harmful to the components of ECM [89]. This is where SCCO_2_ may be able to facilitate infiltration of and potentially synergize with modifier solutions with minimized concentration required to precipitate the same level of decellularization and sterilization efficacy with much less exposure time to such denaturants [54], thereby preserving the ultrastructure and thus the biomechanical properties to the greatest extent.

While SCCO_2_ without excessive additives generally retains the biomechanical integrity of soft tissues as assessed from most endpoints, the effect of SCCO_2_ on the stiffness and Young’s modulus of tissues remains indeterminate. Whilst the present study showed a statistically significant increase in the SCCO_2_-0.1%-SDS group compared to control, Baldini et al. [46], Mazyar et al. [79], and Antons et al. [90] all found reduced stiffness or elastic modulus in tendons treated with SCCO_2_, although the statistically significant finding in the first study might have been partly due to the smaller mean CSA of the tendons allocated to the SCCO_2_ group. On the other hand, Balestrini et al. [72] observed significantly increased elastic modulus in SCCO_2_-treated lung matrices, similar to what we discovered in tendons in this study. Cross-linking of ECM induced by high pressure was proposed as the underlying mechanism, but is confronted by the negative differential scanning calorimetry (DSC) results reported by Hennessey et al. [59] in SCCO_2_-treated porcine aortic valves, even though it was admitted that transient cross-linking could have possibly happened and went undetected by the analyses. The effect of SCCO_2_ on the stiffness of soft tissues is therefore controversial and warrants further investigation.

Moreover, the present study found that gamma-irradiated tendons at 25 kGy showed similar mechanical properties to those of the control tendons. This agrees well with the report of Balsly et al. [39], where patella tendon grafts were tested at 18.3–21.8 kGy as well as 24–28.5 kGy, while others report damage to the collagen and deleterious effects on the mechanical properties [31,35,37,41,42,43,44,47], especially if gamma irradiation treatment is conducted under room temperature instead of with dry ice [36,91]. This highlights the ongoing debate regarding whether the conventional 25 kGy gamma irradiation used for terminal sterilization significantly undermines the in vitro mechanical properties of musculoskeletal allografts [31,35,36,37,38,39,40,41,42,43,44,45,46,47].

The preservation of biomechanical properties by SCCO_2_ was corroborated by the non-mechanical characterization of SCCO_2_-treated samples other than the SCCO_2_-1%-SDS group in the present study, reiterating the gentle character of SCCO_2_ found by many pilot studies [40,46,59,69,72,73,74,75,76,77,78,79]. Previously, Antons et al. [90] also resorted to SEM for ultrastructural examination and found that the orientation, size, and density of collagen fibers in SCCO_2_-treated tendons and skins were generally comparable to those of the native tendons. Dillow et al. [57] have found that SCCO_2_ treatment did not alter the characteristic IR peaks of poly(lactic-co-glycolic) acid, a type of thermally and hydrolytically labile biomaterial analogous to collagenous tissues. On the contrary, both gamma-irradiated and standard-SDS treated tendons sustained disruptions to their ultrastructure and molecular composition to certain extents, as evidenced by alterations in multiple FTIR absorption peaks associated with collagen. Interestingly, Tilley et al. [86] detected a very similar pattern of changes in collagenous scaffolds dissolved in pH 3.3 aqueous solution regarding the reduction in peaks associated with CH_2_ and carbonyl groups, which were attributed to hydroxylation of these groups. Moreover, the present study’s findings of statistically significant increased area of void and separation of collagen fibers histologically in gamma-irradiated specimens echo with the findings of Irani et al. [79] and Bui et al. [78], reaffirming the destructive nature of ionizing irradiation to ECM [32].

The SCCO_2_-SDS hybrid decellularization protocols experimented in this study were formulated with the assumption that SCCO_2_ would demonstrate a comparable degree of synergy with SDS as with PAA [54]. Additionally, PAA has been reported to enhance detergent penetration into tissue [92,93], and while being a potent sterilant, is included in a number of decellularization protocols [80], which made the outlook of combining SDS and PAA-containing NovaKill^TM^ Gen2 for SCCO_2_-facilitated decellularization even more appealing. Nevertheless, even if the biomechanical properties remained intact, the SCCO_2_-0.1%-SDS protocol was not able to thoroughly decellularize tendons under the conditions utilized in this study (i.e., 2 h, 39 °C and 102 bar without agitation by stirrer). Although the reduction in cellularity in SCCO_2_-0.1%-SDS-treated tendons was found to be statistically significant, it is dwarfed by the sheer number of cells that remained in the treated tendons. We initially believed that this ineffective decellularization could be due to the very low concentration and volume of the SDS modifier solution used. It was previously found that 2 mL of NovaKill^TM^ Gen2 (14.1% PAA) added to the SCCO_2_ treatment system only translated into 0.018% effluent PAA concentration, which was 10 times lower than the typical level of PAA tissues are exposed to in conventional treatment [72]. Thus, although a quantitative measure was not used in the present study, the concentration of SDS specimens that were exposed to the SCCO_2_-0.1%-SDS protocol of this study was likely to be low, for the SEM and FTIR changes in the standard-SDS-treated tendons were not manifested in the SCCO_2_-0.1%-SDS-treated ones. Consequently, we hypothesized that a higher concentration of additives or changes to the treatment conditions such as longer treatment time and higher pressure could potentially result in better decellularization outcomes. However, a 10-fold increase in SDS concentration implemented in the SCCO_2_-1%-SDS protocol did not achieve any decrease in cell count while the mechanical integrity and microscopic features of the tendons were demolished, presumably suggesting overexposure to SDS. Therefore, we propose that the use of CO_2_-philic decellularizing agents are likely to be imperative for effective decellularization [94]. Indeed, CO_2_ is non-polar and requires a compatible cosolvent to remove polar molecules such as phospholipids (abundant in cell membranes) and DNA, and Antons et al. [90] did successfully reduce DNA content of the tendons by combining SCCO_2_ with Dehypon^®^ LS-54, a common household and industrial surfactant, without affecting the mechanical integrity or biocompatibility.

Furthermore, dehydration of SCCO_2_-treated tissues observed in this study, as evidenced by the statistically significant decrease in CSA of SCCO_2_-0.1%-SDS-treated tendons, was also reported by Sawada et al. [95], where they successfully extracted cell nuclei from tissue with SCCO_2_-entrained ethanol. Casali et al. [81] were subsequently able to overcome this problem by pre-saturating SCCO_2_ with water.

The present study does suffer from many limitations. The choice of ovine hindlimb digital tendons ensured the availability as each sheep was able to yield a total of 4 viable tendons for mechanical testing. However, the discrepancy in dimensions (Table 1) rendered failure load and stiffness data less interpretable, making a direct comparison of these results with other studies’ inappropriate. Meanwhile, the apparent decrease in CSA due to dehydration might have caused an overestimation of the Young’s modulus and the UTS of the SCCO_2_-0.1%-SDS-treated tendons [81]. Also, the present study primarily focused on failure testing, whereas cyclic testing and suture pull-out strength testing might have better depicted the mode of loading tendons that are subject to in vivo and therefore would have been more clinically relevant [45,96]. Furthermore, sterility verification of the treated samples was not performed in the present study, although the samples were treated using the identical methods (i.e., SCCO_2_ conditions and additives) that have been proven to achieve SAL 10^−6^ in a previous study [54]. It is unlikely, but still possible, that SDS added to the treatment concoction might interact with NovaKill^TM^ Gen2 and downgrade its sterilization efficacy, making it necessary to have two separate treatment steps. As for the assessment of decellularization thoroughness, Crapo et al. [89] proposed an acclaimed three-item standard that entails quantitative measurements of DNA content and fragment length, which however, were not incorporated into the present study.

## 4. Materials and Methods

### 4.1. Sample Procurement and Preparation

120 extensor digital tendons were harvested from the hindlimbs of 20 skeletally mature sheep aged more than 2 years immediately following euthanasia for other ethically approved studies conducted at our institution. Harvested tendons were soaked in PBS and frozen at −60 °C until further treatment and/or testing. All tendons were stored for a maximum of 6 months and underwent no more than 3 freeze–thaw cycles to minimize the effect of repetitive freeze–thaw cycles on their mechanical strength [97,98].

### 4.2. Study Design

**Figure 5:**
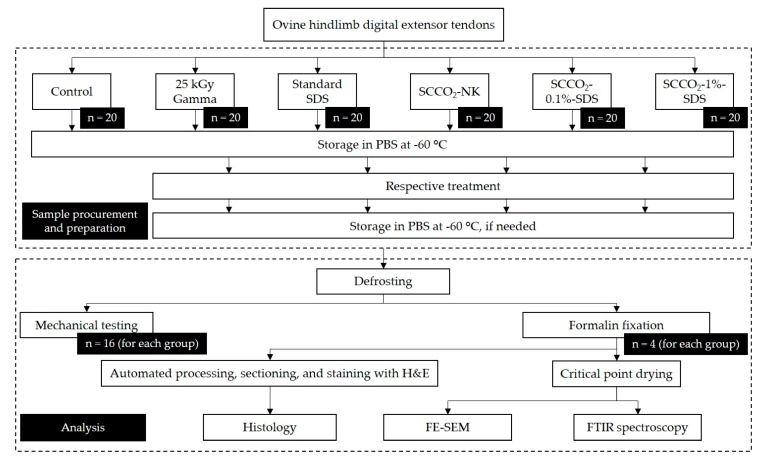
A study design flowchart showing the sample size for each group and endpoint, and processing and storage steps. Abbreviations: SDS, sodium dodecyl sulfate; SCCO_2_, supercritical carbon dioxide; PBS, phosphate-buffered saline; FE-SEM, field-emission scanning electron microscopy; FTIR, Fourier-transform infrared.

6 groups were proposed as previously discussed in the introduction section. Each group was randomly allocated with 16 full-length tendons (approximately 10 mm in length) for mechanical testing and 4-cm tendon segments produced from 4 full-length tendons for non-mechanical assessments (Figure 5). Samples underwent different treatments accordingly, and if not otherwise specified, were soaked in PBS, and either frozen until mechanical testing or fixed in 10% neutral buffered formalin to be prepared for histology, FE-SEM, and FTIR spectroscopy.

### 4.3. Gamma Irradiation Treatment

Samples were packaged in Tyvek sterilization pouches (Livingstone International, Mascot, Australia), sealed in a Styrofoam box filled with dry ice to maintain a temperature below −20 °C throughout treatment, and then transferred to an external treatment facility, where they were irradiated with a cobalt^60^ source at the dose of 25 kGy under standardized operating procedures (Steritech, Wetherill Park, Australia). Treated tendons were then returned to the −60 °C freezer, or thawed and fixed in formalin.

### 4.4. Standard SDS Decellularisation

The standard SDS treatment of the present study was strictly reproduced from the protocol of Pridgen et al. [82]. Briefly, samples were thawed and washed at room temperature on an orbital shaker at 20 rpm with 0.1% (*w/v*) ethylenediaminetetracetic acid (EDTA) (Sigma-Aldrich, St. Louis, MO, USA) in de-ionized water (Merck Millipore, Burlington, MA, USA) for 4 h with a single change of solution at the 2-h mark. The pre-treated samples were then washed in 0.1% (*w/v*) SDS (Sigma-Aldrich, St. Louis, MO, USA) prepared in 0.1% (*w/v*) EDTA for 24 h with a single change at 12 h, followed by PBS wash for 1 h with a single change at 30 min.

### 4.5. SCCO_2_-Based Treatment Protocols

**Figure 6:**
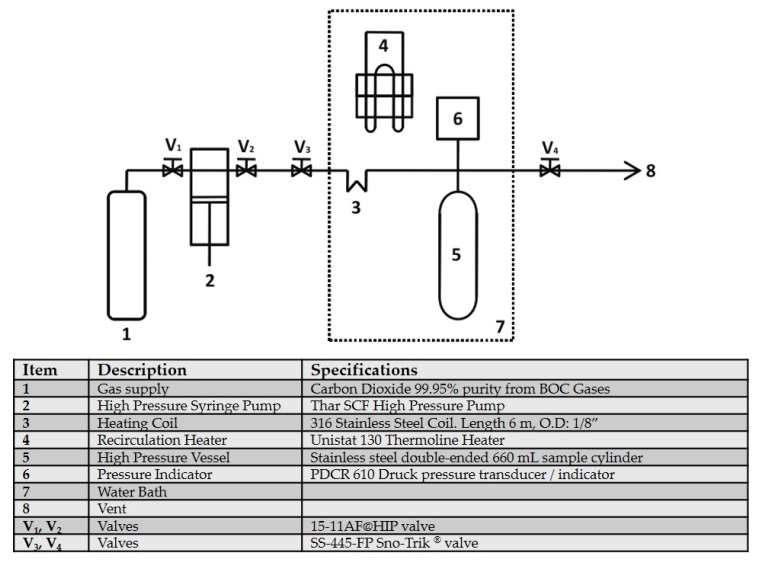
Schematic of the in-house supercritical fluid system [76].

An in-house custom-built supercritical fluid system was used to carry out SCCO_2_ treatment protocols (Figure 6) [76]. A cellulose pad secured with a stainless-steel basket at one end of the 660-mL pressure vessel was soaked in and hence served as the vehicle for additive chemicals. Group IV (SCCO_2_-NK) tendons were thawed, packaged into Tyvek pouches, loaded into the pressure vessel, and treated with SCCO_2_ and 3.2 mL per 30 tendons of NovaKill^TM^ Gen2 (~14.1% PAA and ~4.9% H_2_O_2_) (NovaSterilis, Lansing, NY, USA) under 39 °C and 102 bar to mirror the established SCCO_2_ sterilization protocol that has been proven to achieve SAL 10^−6^ [54]. The treatment vessel was slowly depressurized over 30 min, and the tendons retrieved and either frozen or formalin-fixed until further analysis

Group V (SCCO_2_-0.1%-SDS) and group VI (SCCO_2_-1%-SDS) tendons first received the above-described [82] 4-h pre-treatment with EDTA, followed by 2 h of SCCO_2_ treatment under the identical conditions as group IV tendons. Also, in addition to NovaKill^TM^ Gen2, the additive chemical cocktail was reinforced with 0.8 mL per gram of tissue wet mass of the 0.1% SDS and 0.1% ETDA solution for group V, and 1% SDS and 0.1% ETDA for group VI, with an attempt to decellularize.

### 4.6. Mechanical Testing

**Figure 7:**
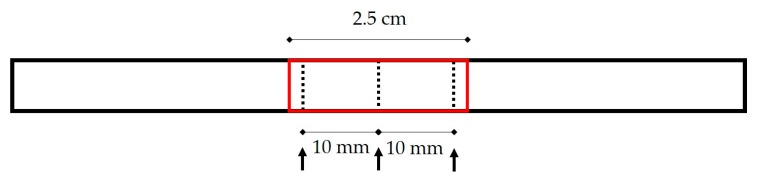
The locations of width and thickness measurements. The bold black frame represents a full-length tendon. The central mid-substance encircled in the red frame was designated as the gauge length for mechanical testing. The dashed lines pointed by black arrows delineate where the width and thickness measurements were taken.

The pre- and post-treatment width (w) and thickness (t) of the tendons were measured with a digital caliper at the center of the mid-substance section intended to be tested and the two points 10 mm away towards each end (Figure 7). The cross-sectional area (CSA) was assumed elliptical and calculated with the formula CSA = π × t × w/4 [42]. The mean CSA of the tested section is then estimated by averaging the calculated CSA at the three equidistant points. Uniaxial tensile testing was conducted by the MTS 858 Mini Bionix^®^ axial servohydraulic tabletop test system (MTS, Eden Prairie, MN, USA) with a 1 kN load cell. Thawed tendons were rigidly griped at both ends by cryo-clamps with interlocking rectangular teeth. The gauge length was set at 25 mm. Testing commenced after a freeze-line was observed at the gripping edge of each clamp, and tendons taut with a 2 N pre-load. The testing protocol was programmed in and executed by the MTS TestSuite^TM^ Multipurpose Elite Software (MTS, Eden Prairie, MN, USA), with force and displacement data collected at 10 Hz. Each tendon was subjected to 10 pretensioned cycles between 10 and 20 N at 1 Hz, stretched to and held at 8% strain for 10 s, ramped back to 0% strain to fully relax, and then ramped at a rate of 100% strain/min to failure, which was defined as a plummet in force profile succeeding the maximum force reached. Testing data were processed by an in-house MATLAB (Mathworks, Natick, MA, USA) script, where the force-displacement and stress-strain graphs were plotted to derive both the mechanical properties, namely failure load and stiffness, and the CSA-normalized material properties, namely ultimate tensile stress (UTS), maximum strain, and Young’s modulus, of the tested tendons for data analysis.

### 4.7. Histology

**Figure 8:**
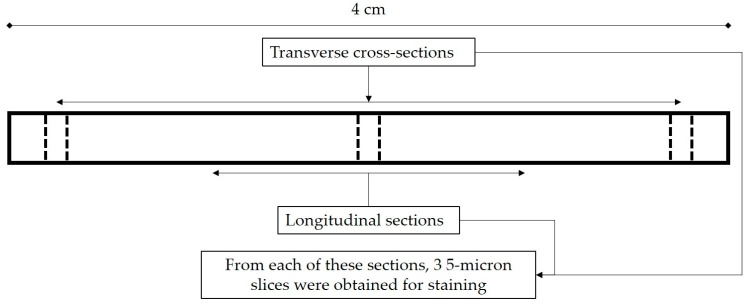
Sample preparation for histology. The bold frame represents a 4-cm tendon segment and dash lines indicate where cuts were made with a scalpel to 3 transverse and 2 longitudinal sections. From each of these sections, 5-micron slices were acquired for staining with haematoxylin and eosin (H&E). Two 40× magnification images were taken for each cross-section, culminating in 18 images per group as there were 3 transverse cross-sections per tendon segment, and 3 tendon segment per group.

Three transverse and two longitudinal sections were obtained along the length of each treated and formalin-fixed 4 cm tendon segment (Figure 8), which were automatically processed with Shandon™ Excelsior™ ES tissue processor (Thermo Fisher Scientific, Kalamazoo, MI, USA) and embedded in paraffin wax with standard procedures. Five-micron slices were then obtained with a hand-operated Leica RM 2165 Microtome (Leica Instrument GmbH, Nussloch, Germany) from each section and stained with haematoxylin and eosin (H&E). An Olympus BX43 microscope was employed in conjunction with a DP72 digital camera (Olympus, Shinjuku, Japan) to thoroughly examine the histological appearance of tendons at both low and high magnifications. The cellularity and percentage of empty space (void) within each snapshot were quantitatively determined with an in-house MATLAB (Mathworks, Natick, MA, USA) script. For each group, the average results of a total of 18 images taken under 400× magnification at random locations of transverse H&E-stained cross-sections (2 per cross-section, 3 cross-sections per tendon segment, and 3 tendon segments per group) were deemed representative of the overall picture.

### 4.8. Scanning Electron Microscopy

Formalin-fixed samples were decanted in deionized water for 15 min, dehydrated with increasing grades of ethanol, critical-point-dried with an Autosamdri-815 Critical Point Dryer (Tousimis, Rockville, MD, USA), mounted onto 12.5-mm sample stubs, sputter-coated with platinum (40 mA, 2 × 2.5 min) using an Emitech K575X Sputter Coater (Quorum Technologies, Lewes, UK), and then examined under the FEI Nova NanoSEM 230 field-emission scanning electron microscope (Thermo Fisher Scientific, Waltham, MA, USA). Snapshots of the longitudinal collagen fibres and tendon surfaces were obtained from 100× to 120,000× magnifications.

### 4.9. Fourier-Transform Infrared Spectroscopy

Following critical point drying, samples from each group were analyzed with a Spectrum Two FT-IR Spectrometer (PerkinElmer, Waltham, MA, USA). The attenuated total reflectance (ATR) mode was used to compensate for the discrepancy in the thickness of the samples. For each specimen, a constant pressure was applied to obtain a consistent absolute absorbance and the spectra were generated from 4000 to 400 cm^−1^ based on 64 background-subtracted scans, and then ATR corrected and smoothed with the PerkinElmer Spectrum software for analysis.

### 4.10. Statistical Analysis

Statistical analysis was performed using IBM SPSS statistics 25 (SPSS Inc., Chicago, IL, USA) for all quantitative endpoints using a one-way analysis of variance (ANOVA) if not otherwise specified. A statistically significant effect in ANOVA was followed by a Tukey’s post hoc test if the homogeneity of variances assumption was met, or if not, Games–Howell post hoc test. A *p* value of less than 0.05 is considered statistically significant. All figures are reported as mean ± standard deviation.

## 5. Conclusions

Development and modification of new allograft tendon and tissue processing protocols, in general, follow a multi-step progression. SCCO_2_ represents an exceptionally promising but under-investigated agent, and the present study has demonstrated preservation of mechanical properties of SCCO_2_-treated tendons. The next advancement would be to identify the optimal CO_2_-philic detergents that complement SCCO_2_, and the treatment parameters to actualize sterilization and decellularization with minimal concentration and protocol run-time. The in vivo biocompatibility of SCCO_2_-treated tendons should then be studied as biomechanical integrity is only one of the many determinants for clinical success. Host cell infiltration and repopulation of implanted tendon allografts, for example, are indispensable for graft incorporation and maintenance against progressive attenuation of mechanical strength with everyday use [99,100]. The capacity of tendon allografts to promote and accommodate cellular ingrowth is affected by treatment and usually limited due to the compact nature of the collagen structure [101,102]. SCCO_2_ has the potential of creating porous scaffolds that are advantageous to cellular attachment [78,103,104], and validating such a possibility is certainly a worthwhile undertaking for future in vivo studies.

## Figures and Tables

**Table 1:** Pre- and post-treatment dimensions.

Group	Cross-Sectional Area (CSA) (mm^2^)		Thickness (mm)		Width (mm)	
	Pre-Treatment	Post-Treatment	Pre-Treatment	Post-Treatment	Pre-Treatment	Post-Treatment
Control	3.75 ± 0.96		1.64 ± 0.21		2.90 ± 0.56	
25 kGy Gamma	4.61 ± 1.32 ^a^	4.34 ± 1.00	1.84 ± 0.29	1.69 ± 0.21	3.15 ± 0.53	3.26 ± 0.62
Std SDS	4.89 ± 0.82 ^a^	4.78 ± 0.89 ^a^	1.85 ± 0.14 ^a^	1.85 ± 0.17 ^ab^	3.39 ± 0.63 ^a^	3.32 ± 0.67 ^a^
SCCO_2_-NK	4.33 ± 0.84	4.14 ± 0.73 ^c^	1.70 ± 0.19	1.70 ± 0.18 ^c^	3.25 ± 0.59	3.06 ± 0.60
SCCO_2_-0.1%-SDS	4.31 ± 0.95	3.53 ± 0.84 ^bcd^*	1.73 ± 0.12	1.62 ± 0.11 ^c^*	3.16 ± 0.66	2.78 ± 0.65 ^bc^
SCCO_2_-1%-SDS	4.05 ± 0.81 ^c^	4.17 ± 0.79 ^ce^	1.73 ± 0.16	1.76 ± 0.22 ^e^	2.97 ± 0.51 ^c^	3.02 ± 0.42

Although the measurements were conducted on the same tendons for each group, the pre- and post-treatment measurements could not be matched. Thus, independent samples t-tests were run to compare the pre- and post-treatment dimensions for each group. ^a^ Significant difference from control in the respective column; ^b^ Significant difference from 25 kGy Gamma in the respective column; ^c^ Significant difference from Std SDS in the respective column; ^d^ Significant difference from SCCO_2_-NK in the respective column; ^e^ Significant difference from SCCO_2_-0.1%-SDS in the respective column; * Significant difference from pre-treatment measurements.

**Table 2:** The mean values of mechanical properties (failure load and stiffness) (a) and material properties (UTS, failure strain, and Young’s modulus) (b) of all groups.

**(a)**	**Failure Load** (**N**)	**Stiffness** (**N/mm**)	
Control	330.34 ± 79.09	77.03 ± 31.83	
25 kGy Gamma	408.27 ± 121.86	92.58 ± 23.95	
Std SDS	350.55 ± 128.77	72.95 ± 29.80	
SCCO_2_-NK	376.79 ± 93.19	98.54 ± 26.22	
SCCO_2_-0.1%-SDS	400.31 ± 123.79	108.65 ± 21.86 ^ac^	
SCCO_2_-1%-SDS	203.07 ± 103.14 ^abcde^	73.09 ± 24.16 ^e^	
(**b**)	**Ultimate Tensile Stress** (**MPa**)	**Failure Strain** (**%**)	**Young’s Modulus** (**MPa**)
Control	89.76 ± 15.40	40.01 ± 10.12	574.20 ± 253.81
25 kGy Gamma	93.88 ± 14.02	40.73 ± 6.90	557.87 ± 135.27
Std SDS	72.68 ± 17.35 ^b^	42.70 ± 9.68	405.43 ± 101.82
SCCO_2_-NK	92.57 ± 23.43	36.68 ± 7.20	648.40 ± 181.71 ^c^
SCCO_2_-0.1%-SDS	112.84 ± 19.84 ^abc^	34.44 ± 5.32 ^c^	852.63 ± 193.67 ^abcd^
SCCO_2_-1%-SDS	50.75 ± 26.27 ^abde^	28.93 ± 5.35 ^abc^	456.55 ± 157.34 ^de^

^a^ Significant difference from control in the respective column; ^b^ Significant difference from 25 kGy Gamma in the respective column; ^c^ Significant difference from Std SDS in the respective column; ^d^ Significant difference from SCCO_2_-NK in the respective column; ^e^ Significant difference from SCCO_2_-0.1%-SDS in the respective column.

**Table 3:** Void percentage and cellularity across groups.

Group	Void (%)	Cellularity (Cells/mm^2^)
Control	29.08 ± 4.96	818.91 ± 116.62
25 kGy Gamma	41.81 ± 4.50 ^a^	750.61 ± 176.45
Std SDS	35.21 ± 4.48 ^ab^	3.17 ± 9.49 ^ab^
SCCO_2_-NK	27.61 ± 6.49 ^bc^	764.65 ± 127.28 ^c^
SCCO_2_-0.1%-SDS	27.35 ± 7.09 ^bc^	682.32 ± 126.42 ^ac^
SCCO_2_-1%-SDS	7.48 ± 5.18 ^abcde^	824.65 ± 252.15 ^c^

^a^ Significant difference from control in the respective column; ^b^ Significant difference from 25 kGy Gamma in the respective column; ^c^ Significant difference from Std SDS in the respective column; ^d^ Significant difference from SCCO_2_-NK in the respective column; ^e^ Significant difference from SCCO_2_-0.1%-SDS in the respective column.

## Abbreviations

ANOVA	Analysis of variance
ATR	Attenuated total reflectance
CSA	Cross-sectional area
ECM	Extracellular matrix
EDTA	Ethylenediaminetetracetic acid
(FE-)SEM	(Field-emission) scanning electron microscopy
FTIR	Fourier-transform infrared (spectroscopy)
H&E	Haematoxylin and eosin
PAA	Peracetic acid
PBS	Phosphate-buffered saline
SAL	Sterility assurance level
SCCO_2_	Supercritical carbon dioxide
SDS	Sodium dodecyl sulfate
UTS	Ultimate tensile stress
